# Urticaria: recommendations from the Italian Society of Allergology, Asthma and Clinical Immunology and the Italian Society of Allergological, Occupational and Environmental Dermatology

**DOI:** 10.1186/s12948-020-00123-8

**Published:** 2020-05-06

**Authors:** Eustachio Nettis, Caterina Foti, Marina Ambrifi, Ilaria Baiardini, Leonardo Bianchi, Alessandro Borghi, Marco Caminati, Giorgio Walter Canonica, Marco Casciaro, Laura Colli, Giselda Colombo, Monica Corazza, Antonio Cristaudo, Giulia De Feo, Ornella De Pita’, Mario Di Gioacchino, Elisabetta Di Leo, Filippo Fassio, Sebastiano Gangemi, Alessia Gatta, Katharina Hansel, Enrico Heffler, Cristoforo Incorvaia, Maddalena Napolitano, Cataldo Patruno, Silvia Peveri, Paolo Daniele Pigatto, Cristina Quecchia, Anna Radice, Giuseppe Alvise Ramirez, Paolo Romita, Franco Rongioletti, Oliviero Rossi, Eleonora Savi, Gianenrico Senna, Massimo Triggiani, Myriam Zucca, Enrico Maggi, Luca Stingeni

**Affiliations:** 1 Department of Emergency and Organ Transplantation, School and Chair of Allergology and Clinical Immunology, University of Bari - Aldo Moro, Bari, Italy; 2 Department of Biomedical Science and Human Oncology, Dermatological Clinic, University of Bari, Bari, Italy; 3 San Gallicano Dermatological Institute -IRCCS, Rome, Italy; 4 Department of Biomedical Sciences, Humanitas University, Pieve Emanuele, Milan, Italy; 5 Section of Dermatology, Department of Medicine, University of Perugia, Perugia, Italy; 6 Section of Dermatology, Department of Medical Sciences, University of Ferrara, Ferrara, Italy; 7 Asthma Center and Allergy Unit, University of Verona and General Hospital, Verona, Italy; 8 Personalized Medicine, Asthma and Allergy, IRCCS Humanitas, Rozzano, Milan, Italy; 9 School and Division of Allergy and Clinical Immunology, Department of Clinical and Experimental Medicine, University of Messina, Messina, Italy; 10 Department of Biomedical, Surgical and Dental Sciences, Clinical Dermatology, IRCCS Galeazzi Orthopaedic Institute, University of Milan, Milan, Italy; 11 Unit of Immunology, Rheumatology Allergy and Rare Diseases, IRCCS Ospedale San Raffaele, Milan, Italy; 12 Division of Allergy and Clinical Immunology, University of Salerno, Salerno, Italy; 13 Clinical Pathology and Immune Inflammatory Disease of the Skin, Cristo Re Hospital, Rome, Italy; 14 Department of Medicine and Science on Ageing, School of Medicine, G. d’Annunzio University, Chieti-Pescara, Italy; 15 Section of Allergy and Clinical Immunology, Unit of Internal Medicine, “F. Miulli” Hospital, Strada Provinciale per Santeramo Km 4.100, Acquaviva Delle Fonti (BA), Italy; 16 Allergy and Clinical Immunology Unit, Azienda USL Toscana Centro, Florence, Italy; 17 Cardiac/Pulmonary Rehabilitation, ASST Pini-CTO, Milan, Italy; 18 Section of Dermatology, Department of Medicine and Health Sciences Vincenzo Tiberio, University of Molise, Campobasso, Italy; 19 Department of Health Sciences, University Magna Graecia of Catanzaro, Catanzaro, Italy; 20 Allergy Department Unit, Piacenza Hospital, Piacenza, Italy; 21 Centro “Io e l’Asma”, Ospedale dei Bambini, ASST Spedali Civili, Brescia, Italy; 22 Immunoallergology Unit, Careggi University Hospital, Florence, Italy; 23 Dermatological Clinic/UC of Dermatology, Department of Medical Science and Public Health, AOU Cagliari, Cagliari, Italy; 24 Department of Experimental and Clinical Medicine and Center of Excellence for Research, Transfer and High Education DENOTHE of the University of Florence, Florence, Italy

**Keywords:** Acute urticaria, Chronic urticaria, Angioedema, Guidelines, Antihistamines, Corticosteroids, Omalizumab

## Abstract

**Background:**

Urticaria is a disorder affecting skin and mucosal tissues characterized by the occurrence of wheals, angioedema or both, the latter defining the urticaria-angioedema syndrome. It is estimated that 12–22% of the general population has suffered at least one subtype of urticaria during life, but only a small percentage (estimated at 7.6–16%) has acute urticaria, because it is usually self-limited and resolves spontaneously without requiring medical attention. This makes likely that its incidence is underestimated. The epidemiological data currently available on chronic urticaria in many cases are deeply discordant and not univocal, but a recent Italian study, based on the consultation of a national registry, reports a prevalence of chronic spontaneous urticaria of 0.02% to 0.4% and an incidence of 0.1–1.5 cases/1000 inhabitants/year.

**Methods:**

We reviewed the recent international guidelines about urticaria and we described a methodologic approach based on classification, pathophysiology, impact on quality of life, diagnosis and prognosis, differential diagnosis and management of all the types of urticaria.

**Conclusions:**

The aim of the present document from the Italian Society of Allergology, Asthma and Clinical Immunology (SIAAIC) and the Italian Society of Allergological, Occupational and Environmental Dermatology (SIDAPA) is to provide updated information to all physicians involved in diagnosis and management of urticaria and angioedema.

## Background

Urticaria is a disorder affecting skin and mucosal tissues characterized by the occurrence of wheals, angioedema or both, the latter defining the urticaria-angioedema syndrome. The wheal is a skin lesion presenting with a central edema of variable size, surrounded by erythema and associated to itching or, more rarely, feeling of warmth, that are transient, with spontaneous resolution in less than 24 h, and with no relics [1]. Under histological point of view, the wheal is characterized by edema of the superficial derma with a slight-moderate dilation of the vessels, in absence of wall damage and leucocytoclasy, with a perivascular granulocytic infiltrate of eosinophils and neutrophils with rare macrophages and lymphocytes. Angioedema is defined by a cutaneous and/or mucous lesion characterized by rapid onset of non-improntable and non-inflammatory edema of the deep dermis or subcutis, associated with pain or, less frequently, itching, with resolution within 72 h. Histologically, edema massively involves the deep dermis and the hypodermis, with a mostly perivasal granulocytic infiltrate. Urticaria must be distinguished from other diseases in which the hives and angioedema, or clinically similar lesions, can present as symptoms. They include anaphylaxis, vasculitic urticaria (corresponding to a leukocytoclastic vasculitis), pigmentary urticaria (corresponding to a form of cutaneous mastocytosis), some cutaneous manifestations of ectoparasites, autoinflammatory syndromes, bradykinin-mediated angioedema (such as for example, hereditary C1-inhibitor deficiency), Gleich’s syndrome (recurrent angioedema with eosinophilia) or Wells syndrome (granulomatous dermatitis with eosinophilia) [1]. Acute urticaria (AU) can occur in all ages, is usually self-limited and resolves spontaneously without requiring medical attention. This makes likely that its incidence is underestimated.

The aim of the present document from the Italian Society of Allergology, Asthma and Clinical Immunology (SIAAIC) and the Italian Society of Allergological, Occupational and Environmental Dermatology (SIDAPA) is to provide updated information to all physicians involved in diagnosis and management of urticaria and angioedema.

## Classification of urticaria

Classification is based on duration of clinical manifestations and on causative agents.

AU is defined by a duration of < 6 weeks while for chronic urticaria (CU) the duration is ≥ 6 weeks. As far as causes are concerned, Table 1 shows the various agents inducing urticaria.

**Table 1:** Types of urticaria and causative agents

Type of urticaria	Causative agent
Allergic	Foods, drugs, Hymenoptera stings
Spontaneous urticaria	Unknown
Inducible urticaria	Dermographism Cold Heat Sunlight Pressure Vibration Contact Colinergic Aquagenic

### Acute urticaria

It is estimated that 12–22% of the general population has suffered at least one subtype of urticaria during life [2–5], but only a small percentage (estimated at 7.6–16%) has AU [6–9]. The age group studied may be important because AU seems more common in very young children, often linked to infections [10]. In the adult population there is a female preponderance (about 60%), while this gender difference is less evident in children [11–13]. AU is classified as idiopathic in 30–50% of cases [7, 14, 15]. An association with respiratory tract infections can be present in children and adults, in the latter being important to distinguish the role of infection from that of the drug to treat it [16, 17]. Drug-induced urticaria, especially concerning Non steroidal anti-inflammatory drugs (NSAIDs), can be commonly observed in elderly people [18]. Overall, drugs are reported as cause of AU in 9.2–27% of cases, antibiotics, NSAIDs and Angiotensin Converting Enzyme (ACE)-inhibitors being reported as most commonly associated to AU [19, 20]. Often the mechanism underlying the reaction is not IgE-mediated. Also, food allergy may be clinically expressed as AU, in children the food most frequently responsible is cow’s milk [8]. Food induced AU is mostly IgE-mediated and thus the symptoms are of the immediate type, occurring from few minutes to 2 h from ingestion. In a variant, AU may develop only when physical exercise is performed 2–3 h after the contact with the causative food.

The diagnosis of urticaria may be complicated by the heterogeneity of its phenotypes. The diagnostic work-up must start with an accurate clinical history defining the trait, the duration in order to distinguish acute from chronic forms, and the possible causative factors [1, 21]. Table 2 summarizes the main information to obtain according to guidelines [22, 23]. The next step is an objective evaluation that assesses the appearance of the elemental lesions (wheals and/or angioedema) and the absence of signs suggestive of an inflammatory nature of urticaria (vasculitis, polymorphic erythema, etc.) In the latter case, especially if the individual lesions persist for more than 24 h, the patient should be referred to dermatological observation. In the absence of appreciable manifestations at the time of the visit it is very useful to reproduce the dermographism, by stroking a blunt tip on the patient’s back, to highlight the appearance of a typical linear stroke detected along the site of mechanical action. Another important aspect is the general state of health and the quality of life (QoL) perceived by patients with chronic urticaria is comparable with that of patients with chronic coronary artery disease. However, a validated QoL questionnaire is currently available and developed exclusively in patients with CU [24, 25]. To establish the urticaria activity, a score can be calculated according to objective assessment of given items, as reported in Table 3. Subsequent diagnostic procedures depend strictly on what is highlighted in the clinical history. In case of short-onset urticaria, if an allergic origin is suspected (from food, drugs, hymenoptera stings), it is important to perform allergy testing with the appropriate techniques and materials, including skin tests, (prick test or intradermal test), specific IgE measurement, provocation test with suspected drugs or foods [26]. When multiple positive tests are observed, the modern techniques of component resolved diagnosis (CRD) are indicated [27], that may discriminate primary (genuine) sensitizations from cross-reactions.

**Table 2:** Questions to assess by history in patients with urticaria

1	Time of the first onset of urticaria
2	Frequency of symptoms and duration of the single wheal
3	Circadian variations
4	Appearance on weekends, holidays or trips abroad
5	Size, shape and distribution of wheals
6	Associated angioedema
7	Concomitant subjective symptoms (itching, burning, pain, etc.)
8	Familiar history of urticaria and atopy
9	Previous or concomitant diseases (allergic, infective, gastroenterological, psychiatric)
10	Surgical implants or events during surgery
11	Potential triggers (physical exercise, physical agents, foods, occasional drugs, etc.)
12	Concomitant medication intake (NSAIDs, vaccines, hormones, laxatives, ear or eye drops, suppositories, natural remedies, etc.)
13	Apparent correlation with given food(s)
14	Correlation with the menstrual cycle
15	Cigarette smoking
16	Kind of work and hobbies
17	Stressful episodes
18	Quality of life related to current symptoms
19	Previous treatments for urticaria and its efficacy

**Table 3:** Urticaria Activity Scale

Score	Wheals	Itching
0	Absent	Absent
1	Mild (< 20 wheals/24 h)	Mild (present but not bothersome)
2	Moderate (20–50 wheals/24 h)	Moderate (bothersome but not interfering with daily activities and sleep)
3	Severe (> 50 wheals/24 h)	Severe (interfering with daily activities and sleep)

In isolated angioedema, particularly when recurring from years and persisting over 24 h despite corticosteroid treatment, a differential diagnosis with hereditary or acquired angioedema must be performed by the level of C4 and the quantitative and functional measurement of C1-inhibitor [28].

### Chronic inducible urticaria

The responsibility of a given causative factor defines the group of chronic inducible urticaria (CIU), which is characterized by the occurrence of wheal or angioedema induced by stimuli including cold, heat, dermographism, pressure, vibration, sunlight and water and represent 20–35% of chronic urticaria. Contact, cholinergic and aquagenic urticaria are not included in this group, because the triggering factor is not physical

There are some characteristics that are shared in the various forms of inducible urticaria:

(1) the clinical manifestation develops only after an adequate stimulus and is regularly reproduced by it; (2) usually the latency time varies from a few minutes (e.g. cold urticaria) to 3–12 h (e.g. pressure urticaria); (3) in general, the single episodes last for about 30–60 min (with the exception of pressure-delayed urticaria) and the symptoms are localized at the stimulus site; (4) after the regression of the lesions without leaving any relic, there is a refractory period that is variable, depending on the nature of the stimulus (but generally lasting 24–48 h), probably linked to the exhaustion of chemical mediators [21, 29]; (5) the coexistence of two or more forms of inducible urticaria is quite common in the same patient [6, 30, 31].

In diagnostic work-up, the aspects of critical importance are clinical history, because the suspicion of an inducible urticaria can arise from the location, distribution and morphology of the lesions, in relation to a specific triggering factor, and the provocation test, that allows us to confirm the diagnosis and to identify the stimulus capable to elicit urticaria, followed by the threshold test that allows the identification of an individual threshold of reactivity to the stimulus [32]. The knowledge of the stimulus threshold is useful to the patient for the prevention of urticaria and to the physician for the evaluation of the activity of the disease and for the monitoring of the therapeutic responses. The provocation test should be done before and during therapy. In this regard it is useful to remember that before performing the test it is necessary to stop any therapies in place (antihistamines should be suspended at least 3 days before the test and systemic corticosteroids at least 7 days before). Furthermore, it should be noted that, if a period of refractoriness persists after an urticaria manifestation, the provocation test should be carried out at least 24 h after the last episode of urticaria. Patients for whom there is a strong suspicion of an inducible urticaria but who have a negative provocation test should be retested on skin free from urticaria for at least 3 days. It should be emphasized, however, that in some patients, despite the presence of a highly suggestive clinical history for CIU, the provocation test could give a negative result. Moreover, since there is the possibility of coexistence in the same subject of multiple subtypes of CIU, the provocation test should be performed by examining all the stimuli that are suspected to be implicated in the patient’s CIU [32]. Table 4 summarizes the main features of provocation tests.

**Table 4:** Main provocations tests used for diagnosis of inducible urticaria

Kind of urticaria	Test site	Execution method	Reading time	Positive response^a^
Cold urticaria	Volar surface of the forearm	Contact with a ice cube in a thin plastic bag for 5 min	10′	Localized wheal
Heat urticaria	Volar surface of the forearm	Contact with a cylindrical container filled with hot water for 5 min	10′	Localized wheal
Sunlight urticaria	Buttocks	Irradiation with 6 J/cm^2^ of UVA and 60 mJ/cm^2^ of UVB	10′	Localized wheal
Vibratory angioedema	Volar surface of the forearm	Contact with a flat surface placed on a laboratory vortex at a speed between 780 and 1380 rpm (average, 1000 rpm) for 5 min	10′	Changes in the circumference of the forearm in 3 points (wrist, central part of the forearm and proximity of the elbow) before and after the stimulus
Colinergic urticaria		Physical exercise (stationary bike or treadmill) until sweating or until the appearance of skin symptoms	Immediate and after 10′	Widespread small wheals
Aquagenic urticaria	Side surface of the neck Upper part of the back	Contact with tablets soaked in warm water for up to 40 min for 5 min	Immediate and after 10′	Localized small wheals
Contact urticaria^b^	Volar surface of the forearm	Contact with the suspected agent for 30′ (*open test*) If negative, *patch test* *scratch patch test* *prick test* *specific IgE in serum*	Immediate and up to 60′	Localized wheal

^a^The positive response to the test can rarely vary in terms of location and morphology; possible but rare diffuse or systemic reactions

^b^Avoid performing the test in case of a consistent medical history, in particular if associated with systemic symptoms

### Chronic spontaneous urticaria

The epidemiological data currently available on CU in many cases are deeply discordant and not univocal. The main factors involved in the development of this complex framework are the heterogeneity of the studied populations and of the degree of correspondence between the characteristics of each patient sample and those of the general reference population, the heterogeneity of the diagnostic criteria used [21, 33], and the discrepancies between studies conducted within or outside tertiary referral centers [34] for the diagnosis and therapy of immunological and/or dermatological diseases. Therefore, if recent estimates suggest that the prevalence of CU in the general population is 0.5–1% [35], there is no definite data on the prevalence and annual incidence of spontaneous CU (which includes about 50–75% of cases of CU [4, 36–39]. A recent Italian study, based on the consultation of a national registry, which uses the ICD-9-CM classification system (International Classification of Diseases, 9th Revision, Clinical Modification), reports a prevalence of chronic spontaneous urticaria (CSU) of 0.02% to 0.4% and an incidence of 0.1–1.5 cases/1000 inhabitants/year [40].

CSU is more common in females (72.7% of cases, according to the ASSURE-CSU study, still ongoing [41]), which is comparable to that observed in most autoimmune diseases [35, 41, 42]. Not surprisingly, the autoimmune pathogenesis forms make up about 50% of the total number of cases of CSU [43]. The average age of onset is in the fourth-fifth decade of life, although all age groups can be affected [4, 41]. Unlike other immune-mediated pathologies, CSU resolves over 6 months in 50–71% of patients [4, 38]. However, in 10% of cases it may persist for years and require the use of more aggressive therapeutic regimens [38, 44]. As to pathogenesis, CSU is considered a multifactorial pathology, into which endogenous and exogenous factors contribute. Among the endogenous factors some predisposing Human Leukocyte Antigens (HLA)s have been identified, such as the HLA allele DRB1* 04 (coding for HLA DR4) and the allele DQB1* 0302 (coding for HLA DQ8) [45]. Among exogenous factors, it is known that some bacterial infections (e.g. *Helicobacter pylori*, *Mycoplasma pneumoniae*) or parasitic infections (e.g. *Giardia lamblia, Anisakis simplex*) are associated with the development of CSU. Some foods (particularly alcohol, spicy foods) and drugs (e.g. NSAIDs) or even physical or psychic stress, can be identified as causative agents and triggers of urticarial eruptions. However, in a high percentage of cases (55–70%), CSU is defined as idiopathic because the causes remain unknown [46]. A particular variant of CSU is represented by the “chronic autoimmune urticaria”. This form is defined by the presence in serum of IgG autoantibodies directed against the α-subunit of the high affinity receptor for the crystallizable fragment of IgE (anti-FcεRIα) or, more rarely, IgG anti-IgE. These autoantibodies were found in 35–40% and 5–10% of patients with CSU, respectively [45]. The possibility that some subtypes of CSU may recognize an autoimmune pathogenesis is supported by association with other autoimmune diseases (e.g. rheumatoid arthritis, vitiligo, type 1 diabetes mellitus, Hashimoto’s thyroiditis), from the combination with the HLA DR4 haplotype (which predisposes to diseases such as rheumatoid arthritis and autoimmune thyroiditis) and the good response of some patients to immunosuppressive therapies [42]. Patients with CSU often have non-organ-specific markers of autoimmune disease, such as rheumatoid factor positivity and the presence of antinuclear antibodies [45, 46]. Some authors have demonstrated the formation of direct IgE against autoantibodies such as, in particular, thyroperoxidase (TPO) and the native DNA (dsDNA). In these cases, the autoantigen would induce the activation of mast cells and basophils according to the classic activation mechanism. Approximately one-third of patients with CSU have circulating functional autoantibodies. These antibodies induce the release of mediators by tissue mast cells, in particular the cutaneous ones, and by circulating basophils by cross-linking the FcεRI receptors. BHRA (Basophil Histamine Release Assay) is currently the gold standard for the identification of functional antibodies; however, this method is rather complex, thus the Autologous Serum Skin Test (ASST) is currently used as the best in vivo test for the identification of autoantibodies (sensitivity: 70%, specificity: 80%). ASST also correlates with disease activity [47].

## The pathophysiology of urticaria

Multiple chemical mediators are involved in urticaria, often interacting each other in a complex molecular network. Chemical mediators can be secreted at the systemic or local level following the activation of several cells that are resident in the dermis (mast cells) or recruited from peripheral blood (basophils, eosinophils and other blood cells).

Mast cells have a key pathogenetic role and can be activated by different mechanisms [46]. The most well-known activation mechanism is the contact with an agent that induces a hypersensitivity reaction of the I type with production of IgE that binds to FcεRI receptors.

A new exposure to the trigger factor induces the receptor cross-linking and the activation of the intracellular signaling resulting in mediator’s production. Such mechanism can occur in the absence of re-exposure to the antigen in the presence of anti-FcεRIα or anti-IgE IgG, with a special role for IgG activating the mast cells, that belong mainly to the IgG1 and IgG3 subclasses, able to trigger the complement cascade with production of C5a. This molecule is able to amplify the mast cells response by binding to specific receptors present on their surface. Immunologically activated mast cells quickly release various preformed mediators, including histamine and some proteases (triptases, kinases and carboxypeptidases A), as well as synthesize and release lipid metabolites such as prostaglandins (PG)D2, leukotrienes ((LT)C4 and LTD4) and Platelet Activating Factor (PAF), and, ultimately, cytokines and chemokines, particularly tumor necrosis factor (TNF) α, interleukin (IL)-1, IL-4, IL-6 and IL-13. The activation of mast cells can be done by non-canonical way through molecules known as “superallergens”. Superallergens are proteins of bacterial or viral origin (*Staphylococcus A* protein, *Peptostreptococcus L* protein, HIV gp-120) or endogenous proteins synthesized by the liver during viral infection. These proteins are able to bind non-specifically to surface basal cells IgE interacting with a variable region of heavy chains. The binding of superallergens with mast cell IgE induces the activation of these cells and the secretion of chemical mediators in a way that is similar to that induced by the classic allergens [48].

The pathogenic role of basophils is not yet fully clarified. Some studies demonstrated a significant decrease of the number of basophils in peripheral blood of patients with CU in active phase and a correlation between the basophils decrease and the severity of symptoms [45]. Basophils of patients with CSU express higher levels of substance P, a neuropeptide closely associated with the development of vasodilation and pruritus [49]. Basophils in CSU not only show changes in numbers, but also have an altered function [50]. Basophils from patients with CSU are less responsive to activation with anti-IgE antibodies and C5a. This is probably the result of a receptor desensitization process that occurs particularly in patients in whom the presence of auto-antibodies has been demonstrated.

Also eosinophils, despite they are frequently observed on biopsies from patients with CSU, have an uncertain role. The observation that many patients with CSU present serum antibodies directed against the low affinity receptor, which is present at high concentrations on eosinophils (anti-FcεRII/CD23) supports their pathogenic role. These antibodies induce the release of major basic protein (MBP) which activates mast cells and basophils [50]. Eosinophils also represent the main cell responsible for the activation of the coagulation cascade in patients with CSU as the primary source of tissue factor (TF), which in turn would activate mast cells by binding to the protease activating receptor-2 (PAR-2 receptor). It has been observed that patients with CSU have higher mean levels of factor VIIa, D-dimer, F1 + 2 fragment and fibrin degradation products than controls and that these levels correlate with disease severity [51]. Thrombin levels are also increased, but there is no greater tendency to thrombotic events or alteration of coagulation parameters. Thrombin would activate mast cells by binding to the PAR-1 receptor [52].

Concerning other cells, in lesion biopsies a perivenular infiltrate of lymphocytes is generally found, predominantly CD4+. Cytokines of T helper 2 origin were found in the lesions, such as IL-33 and IL-25. High levels of IL-4 were detected in the serum of patients with CSU. The lymphocyte response in the CSU cannot be attributed to a specific type T helper (Th)1 or Th2 and some authors assume that the lymphocytes infiltrating the lesions actually have a Th0 profile [53]. Increased levels of IL-17, IL-23 and TNFα were also found in serum of patients with CSU, demonstrating a possible pathogenic role of Th lymphocytes 17 [54]. Finally, among the cells involved in the pathogenesis of CSU, platelets could play a key role in the sequence of events involved in both inflammatory and coagulation processes. Some studies claim that the increase in their number, mean corpuscular volume, and some activation/aggregation parameters would be directly correlated with the clinical severity and the degree of autoreactivity [55].

In any case, the pathogenic mechanisms described above have as final results the release of mediators, with a primary role for histamine, which is the main mediator responsible for increased vascular permeability. There are four subtypes of histamine receptors, all of type G protein-coupled receptor (GPCR), but H1 seems to be the most important in the pathogenesis of urticaria. The mechanism of action sees the activation of the G Protein αq pathway and therefore of phospholipase C, which causes an increase in the concentration of Ca2+ cytosolic in the vessels of the dermis. The latter activates the MLCK (Ca2+/calmodulin (CaM)—dependent Myosin Light Chain Kinase) triggering the contraction of the actin-myosin system with cell retraction. The reactions triggered downstream of the H1 receptor are numerous and, in particular in the endothelial cells, there is an activation of several MAP Kinases, all determining an alteration of the system of intercellular junctions (tight and adherens junctions), with gaps formation and consequent increase in vessel permeability. The amount of histamine present in the lesions correlates with the disease activity. However, histamine is not the only mediator involved in the pathogenesis of CSU. Serum tryptase levels were higher in patients with CSU compared to controls both in phase of quiescence and in acute phase [56]. Some studies have focused attention on the endothelium and on the expression of vasoactive molecules and endothelial adhesion.

In the serum of patients with CSU, higher levels of VCAM1, ICAM1 and CCL5/RANTES have been found: these molecules could have the role of markers of endothelial dysfunction and be implicated in the pathogenesis of CSU. However, their serum levels do not seem to correlate with disease activity nor with the severity of the manifestations [57].

Recently, the Vascular Endothelial Growth Factor (VEGF) and the Calcitonin Gene-Related Peptide (CGRP) have been observed in the lesions of the patients with the highest levels of vasodilatory factors [58, 59]. Of interest, VEGF is also an important vasodilator mediator, through the production of nitric oxide (NO). VEGF levels are increased in the plasma of patients with CSU and these levels correlate with disease severity.

Some metalloproteinases (MMP-9) and extracellular matrix degradation products (Endostatin and Thrombospondin 1) are detectable in sera from patients with CSU at higher concentrations than controls. IL-31 is a cytokine produced by different cell types, which has been proposed to be involved in various chronic skin diseases (atopic dermatitis, allergic contact dermatitis, nodular prurigo. IL-31 would also play an important role in the pathogenesis of CSU, particularly in determining pruritus. This symptom represents in some patients the primum movens for the appearance of hives in the forms of dermographic urticaria [60].

Bradykinin is a mediator with potent vasodilating and vasopermeabilizing action, produced by the high molecular weight quininogen due to the action of callicrein. The cellular effects of bradykinin are mediated by the activation of two receptors (B1 and B2) capable of activating nitric oxide synthase with release of NO. The role of bradykinin in the pathogenesis of CSU has not yet been sufficiently investigated, although it is known its effect of induction of wheal after subcutaneous injection and its pathogenic role in various inflammatory diseases of allergic interest (angioedema, rhinitis, asthma) [61].

Still largely unknown are several plasma factors, often generically identified as Histamine Releasing Factors (HRF). The HRFs include both high molecular weight molecules (probably immunoglobulins) and low molecular weight molecules (< 30kD) capable of activating mast cells bypassing the classical activation path represented by the FcεRI-IgE axis [62].

## Impact of urticaria on quality of life

The available literature shows that urticaria is more than a troublesome symptom. Patients with urticaria, when compared to healthy subjects or patients suffering from other diseases, have a significantly reduced health related quality of life (HRQoL) score. Such score, as expected, is lower than in healthy subjects, regardless of age, duration of disease, or the presence or absence of angioedema [63]. However, several studies have confirmed that urticaria significantly affects the well-being and HRQoL of patients [63, 64]. In particular, a study compared the HRQoL of patients with CU with that of patients with coronary ischemic disease waiting for by-pass Surprisingly, despite more severe limitations in mobility and pain in patients with ischemia, in the items related to energy, social isolation and emotional reactions, the scores of the two groups were almost overlapping [65]. Globally, emerges a picture that highlights that this pathology has an impact comparable to more serious diseases. In addition, personal satisfaction levels are reduced concerning sleep, eating behavior, stress resistance, mood, self-esteem, job type and professional role [63]. HRQoL in urticaria can be assessed by patient reported outcomes (PROs), and in particular by validated questionnaire, which include generic questionnaires (aimed at evaluating the state of health in general, thus allowing the comparison between populations of patients with different diseases) and specific questionnaires (developed taking into account the peculiar characteristics of a given clinical condition). The most commonly used generic questionnaires are the Nottingham Health Profile [24] and the Medical Outcomes Study SF-36 [63]. Due to their characteristics, these tools allow comparisons between patients suffering from different diseases and between patients and healthy subjects, but they are not suitable for detecting specific problems related to urticaria. The only specific questionnaire for urticaria is the CU Quality of Life Questionnaire (CU-Q2oL) [25], developed and validated in Italian, and now available in several other languages, which is recommended by the guidelines EAACI/GA2LEN/EDF/WAO for the evaluation of HRQoL in urticaria [21]. Recently, starting from CUQ2oL, a specific tool was developed for the evaluation of HRQoL for single patient use, the CU Patient Perspective (CUPP) [64]. It is composed of 10 questions, structured to make easy the score calculation, and has an indication of the clinical significance of the result obtained. The validation process demonstrated how the CUPP meets the criteria of validity and reliability, which for questionnaires to be used in the individual patient, are much more stringent than those established for the tools developed for population studies.

## Diagnosis and prognosis

Based on the variety of urticaria types, an accurate history collection and a general objective examination constitute a valid starting point [66]. The use of laboratory tests should be directed to the type of urticaria according to a level from 1 to 3 (Table 5). In some forms of urticaria, more specific laboratory tests could be useful [33, 39]. The tests for inducible urticaria were already shown in Table 4. Further diagnostic investigations should only be considered in subjects suffering from a severe and persistent form of urticaria present from prolonged time [67–71].

**Table 5:** Laboratory examination

First level	Blood count; Kidney function Liver function Indices of inflammation Total serum IgE Screening thyroid hormones and autoantibodies to thyroid
Second level (only if history is indicative)	Parasitological examination of stools (3 samples) Autoimmunity Autologous serum skin test Anti-*Helicobacter pylori* antibodies Skin prick test or specific IgE to *Anisakis simplex*
Third level	D-Dimer dosage and assessment of coagulation Assessment of complement Measurement of antibodies to viral agents (EBV, HBV, HCV, CMV…)

Once the patient’s medical history has been collected, it will be useful to carry out a general objective examination with attention to the dermographism (after antihistamine treatment has been withdraw from some days). Carrying out extensive and costly screening protocols is not recommended given the extremely varied nature of the triggering causes. For example, immunoreactions of the first type (such as food allergy) rarely cause chronic urticaria; sometimes the urticaria symptoms could be worsened by a toxic effect of some foods, and then a diet poor in histamine could give relief to the patient [72]. Also, bacterial, parasitic, fungal and viral infections have sometimes been associated with the appearance of pomfoidal lesions (*Helicobacter pylori*, *Streptococcus*, *Staphylococcus*, *Mycoplasma pneumoniae*, *Salmonella*, *Brucella*, *Borrelia*, *Chlamydia, Yersinia enterocolitica, herpesviridaae, parvoviridae, caliciviridae, picornaviridae, flaviviridae, hepadnaviridae, Anisakis simplex*, etc. [65, 72, 73]. A parasitological examination of feces on 3 samples, eosinophil count, total IgE assay, anti-*Helicobacter pylori* antibodies can be useful, as well as a prick test for *Anisakis simplex* and the search for IgM to the above-mentioned pathogens [65, 73, 74]. Sometimes the foci of pathogens can be occult and provide a chronic stimulus (e.g. dental diseases). On the other hand, screening for neoplastic diseases is no longer recommended. A screening test demonstrating the presence of autoantibodies against the high affinity receptor for IgE should be performed (ASST) [75].

Finally, since the coagulation cascade is activated in both self-reactive and non-reactive patients, it is of interest that high levels of D-dimer can identify patients with more severe pathology; to this end, it would be useful to add to the D-dimer assay also an evaluation of the F1 + 2 fragment of prothrombin, of fibrin and fibrinogen degradation products and of the soluble fibrin monomeric complex [76–78].

As to prognosis, CSU is a transitory condition (average duration 2 to 5 years) and does not endanger the patient’s life, even if it is experienced as a disabling condition [79]. In most cases it resolves within a year from the onset of symptoms and only around 10% of patients are affected by the disease over 5 years [80, 81]. In rare cases it goes beyond [82]. However, there are few predictors of response, including genetic factors related to mast cell response, hyper-production of histamine and leukotrienes [83]. The presence of angioedema, severe forms of CU and the presence of antithyroid antibodies have been associated with a longer duration of the disease [84].

## Differential diagnosis of urticaria

While diagnostic tests of standardized provocation are available for the diagnosis of inducible urticaria in accordance with international recommendations, the spontaneous form should be differentiated from vasculitic urticaria, auto-inflammatory syndromes and other forms of urticarial dermatitis based on clinical features, morphological and histopathological. In chronic spontaneous urticaria, the wheals have an asymmetric distribution, they resolve without dyscromic outcome and are associated in about 40% of cases with angioedema.

The urticarioid syndromes, on the other hand, are extremely heterogeneous and may be localized only in the skin or be systemic, but in both cases the clinical pictures are characterized by the presence of atypical, persistent wheals, with bilateral and symmetrical distribution, which resolve with residual discolouration, mostly associated to other lesions such as papules, vesicles, or bubbles rarely associated with angioedema [21].

The most important differential diagnosis of spontaneous CU is with vasculitic urticaria, which represents 5–10% of CU and is characterized by skin lesions similar to those of spontaneous urticaria but with histopathological characteristics typical of cutaneous necrotizing vasculitis [85]. Vasculitis concerns the small vessels, particularly post-capillary venules and there is a form with mainly cutaneous involvement called cutaneous small vessel vasculitis (CSVV). UV (vasculitic urticaria) that can be normocomplementemic (NUV) or hypocomplementemic (HUV) is distinguished by decrease of C1q, C4 and C3 and HUVS or systemic vasculitic urticaria which represents the most severe form of HUV [86]. The diagnosis of HUVS is defined by two major criteria: presence of urticaria, angioedema or both for at least 6 months and hypocomplementemia, and two of the following minor criteria: dermal venulitis, arthralgia, moderate glomerulonephritis, uveitis or episcleritis, recurrent abdominal pain. In vasculitic urticaria, the wheals have different shape and size, widespread to the whole cutaneous area without a preferential localization, and they persist for more than 24 h with itch, often burning or pain, but sometimes asymptomatic. The erythema is intense, it can regress to diascopy highlighting at the center of the lesion petechiae punctuated and ecchymotic shades [85, 86].

Vasculitic urticaria is mostly idiopathic but sometimes drugs such as anti-folate, antidepressants, cimetidine, or physical stimuli like cold, and sun exposure are involved. It may present as a manifestation of a systemic pathology and in particular of connective pathologies such as systemic lupus erythematosus (SLE), paraneoplastic dermatomyositis, systemic sclerosis, or be part of systemic vasculitis such as Wegener’s granulomatosis or Churg-Strauss syndrome [87]. Moreover, it can follow infections such as acute or chronic viral hepatitis and, in some cases, it may be the paraneoplastic manifestation of hemolymphopathies such as non-Hodgkin B cell lymphoma or myeloma [86].

The pathogenetic mechanism underlying the pathology is the formation, by antigens currently unknown, of antigen–antibody complexes that are deposited on the vessel wall, trigger the complementary cascade with subsequent activation of the mast cells, recruitment of neutrophils and vascular damage. Cutaneous biopsy and histological examination with direct immunofluorescence are necessary for diagnosis.

If a patient with recurrent wheals reports osteo-articular pain, unexplained fever and general malaise an auto-inflammatory syndrome, that may be hereditary or acquired, should be suspected. Auto-inflammatory syndromes are a group of monogenic disorders in the genes that regulate the innate immune response, resulting in an aberrant activation of mediators and in particular the IL-1 pathway. Most of them are associated with rash, fever in some cases periodic, but may be unpredictable over time without evidence of neoplastic tumor or autoimmune diseases, arthritis, elevation of inflammation indices, abdominal pain, amyloidosis, myalgias and neurological signs. A detailed description of these syndromes goes beyond our objectives and will be limited to the forms in which urticaria lesions such as cryopyrin-associated periodic syndrome (CAPS) and Mediterranean family fever are more frequently present. CAPS are a group of inherited autoinflammatory syndromes that represent the clinical spectrum of different mutations of the CIAS1 gene (cold induced autoinflammatory syndrome) that encodes a protein called cryopirine (NALP3), a component of the inflammatory agent that activates IL-1alpha when the cell receives the danger signal. CAPS include the Muckle-Wells syndrome (with urticaria, amiloidosis and deafness), the chronic infant with neurologic, cutaneous and articular (CINCA) involvement, the autoinflammatory cooling syndrome with papillary orticarioid lesions after cold exposure accompanied by systemic symptoms and with a negative ice cube trigger test [86].

The Mediterranean family fever affects the populations originating in the Mediterranean more frequently and is associated with a mutant pirine with functional impairment and activation of IL-1beta. The urticaria lesions present in this syndrome are persistent, often bilateral, symmetrical, but there may also be macular, papulose and purpuric lesions accompanied by burning and always with systemic symptoms. [86].

Orticarioid dermatitis was recently defined to describe patients with a peculiar pattern of hyperreactivity. It mostly affects adult patients aged 60 years of age or older, with urticaria and eczematous lesions associated with intense, bilateral and symmetrical pruritus with localization to the trunk and root of the limbs and have a duration of even weeks. Histological examination shows an eczematous reaction with minimal epidermal spongiosis, papillary dermal edema and a superficial perivascular infiltrate of lymphocytes with eosinophils. The etiology is uncertain even though in some cases it has been attributed to a reaction to drugs, antihistamines are not effective, and a short corticosteroid treatment leads to non-definitive remission since the dermatitis can relapse [87].

Drug exanthematic reactions are frequent, occurring after a few days. Although a maculopapulotic rash with erythema initially on the trunk and upper limbs is the most frequent form, the lesions may also be intense with red, persistent orticarioid lesions with bilateral and symmetrical localization and tendency to confluence and residual pigmentation associated with mild itching [87].

Cutaneous mastocytosis, previously called urticaria pigmentosa, is the most common clinical picture of mastocytosis that can mimic urticaria. It concerns pediatric or adult patients with red-brownish macules and papules which become apparent with rubbing (Darier’s sign), with the main site of the trunk and limbs. In pediatric age there may be a urticaria rash with persistent lesions also localized in the face, spontaneous or caused by rubbing, heat or sun exposure. The number of injuries is variable but does not correlate with the risk of systemic involvement. In half of the patients flushing appears and syncopal or anaphylactic episodes can occur. In adults, rarely in children, there may be an asymptomatic involvement of the bone marrow. The diagnosis is clinical and histological with possible recourse to immunohistochemistry for the count of the mast cells. The serum tryptase assay (< 20 ng/ml) is the most important parameter for screening patients with possible systemic involvement and for their follow-up. The prognosis depends on the age of onset and is better for pediatric forms while in adults it is possible an evolution towards a systemic mastocytosis in a percentage ranging from 3 to 30%. Also, in the systemic form there may be episodes of urticaria-angioedema but always associated with other systemic symptoms of the gastro-intestinal, respiratory, cardiological system [88].

In conclusion, urticaria lesions may appear in a variety of skin or systemic diseases.

The clinical-evolutionary criterion is fundamental for the differential diagnosis since the lesions of the urticaria syndromes are predominantly bilateral, symmetrical, are persistent and often resolve with dischromic outcomes, are often associated with different skin lesions and systemic symptoms.

The clinical-pathological correlation with biopsy and histological examination is the other element that is necessary and in some cases indispensable for a correct classification.

## Management of urticaria

The management of urticaria must be faced considering in each patient essentially two aspects, the first related to the identification and elimination of the causes and triggers, the second concerning the effective treatment of symptoms. To treat the patient by avoiding the triggering cause would be the best option, but often in CSU this is not possible because the triggering factor is not recognizable, while it may be possible in the rare cases of IgE-mediated urticaria and partly in the forms induced by physical stimuli. The clinical classification in the various subtypes is important for a correct diagnostic and therapeutic approach. The therapy has the role of reducing the release of mediators and the effect that these exert on the target organs and to induce tolerance.

The severity of clinical manifestations and the nature and strength of the stimulus, which causes and perpetuates the symptoms, are different in individual patients and, for example, it is always necessary to evaluate the environmental situation in the inducible forms, such as hot or cold [21, 89].

### Identification and elimination of triggers

Identifying the causes is not easy, because often more stimuli can be associated in the same patient. In case of remission after the elimination removal of the suspect agent, only the recurrence of symptoms in the presence of the same agent can lead to the recognition of the cause, thus excluding an accidental factor.

Among drugs, antibiotics and NSAIDs are often considered to cause urticaria. When their responsibility is suspected, they must be avoided or replaced to prove their actual responsibility. Drugs may also aggravate pre-existing urticaria [90]. Concerning infections, the most frequently suspected are dental and urinary infections, but there is no evidence on their role; as to *Helicobacter pylori*, contrasting data were reported about the possibility to resolve urticaria by effective treatment of the infection [91].

Food-IgE mediated allergy is rarely associated with chronic urticaria, but if a food allergen is identified as the triggering factor, it must be eliminated from the diet. In some patients there may be a reaction related to non-IgE mediated allergy, but to a hypersensitivity reaction to some foods or ingredients, especially additives [92]. The new European guidelines show that there is evidence of improvement with the removal of pseudo-allergens for periods of at least 3–6 months and how the benefit of this diet on clinical manifestations begins to emerge only after 3 weeks. The benefits achieved are also linked to the type of eating habits and the differences between the various European countries [1, 92, 93].

It is recognized that stressful events can trigger or aggravate urticaria, and that urticaria can worsen a pre-existing stress. Psychological support therapies can be considered in addition to the treatment of the disease [94].

### Pharmacologic treatment of acute urticaria

The therapy of AU and/or angioedema, although strongly conditioned by the identification of the cause, is predominantly symptomatic. The aim of this treatment is to reduce the clinical effects of mediators released by mast cells and mainly of histamine, using drugs capable to exert a competitive blockade of histamine H1 receptors. Second generation antihistamines (SGA) are generally considered to be first line drugs in both acute and CU [21, 95]. Compared with 1^st^ generation anti-histamines, they also have anti-inflammatory effects by inhibiting the release of cytokines from mast cells and basophils [96], have less rapid action but longer half-life (15–20 h) and surpass only minimally the blood–brain barrier having greater molecular weight, lower liposolubility and greater affinity for glycoprotein P. Therefore, the possibility of inducing anticholinergic effects (mucosal dryness, constipation, reduction of diuresis), and sedatives effects is significantly lower, even in the elderly with cognitive disorders, increased intraocular pressure and benign prostatic hypertrophy. Likewise, the frequency of sedative effects from interaction with other drugs (such as analgesics, hypnotics, anxiolytics, antidepressants) and alcohol is lower. SGA (except cetirizine, fexofenadine, bilastine, acrivastine) are metabolized in the liver by cytochrome P450. Thus, the simultaneous administration of other drugs using the same enzymatic system (such as ketoconazole, erythromycin, clarithromycin, rifampicin, itraconazole, cimetidine, cyclosporine) can slow the metabolism of antihistamines and increase their plasma concentration [97].

H1 antihistamines of 1st generation, however, can be used in patients with anxiousness or with insomnia due to nocturnal exacerbations of the skin symptomatology or in the onset phases of urticaria. Taking antihistamines should be regular (not as needed) and should be prolonged for at least 7–10 days after symptom remission.

In cases of inadequate therapeutic response to antihistamines, two different strategies are possible, i.e. increasing the dosage of the antihistamine used up to four times the conventional daily dose or the addition of a corticosteroid. The first approach [21, 98–100] is not always effective, but is generally well tolerated, except for a possible increase in drowsiness. It is however advisable to obtain informed consent from patients.

The association with a corticosteroid to ensure greater control of mediators can be considered in the most serious cases when the wheals are very large and widespread and/or associated with angioedema, and in presence of exacerbation of symptoms [21, 101]. In angioedema, however, the association is not useful when the symptomatology is due to the release of non-mast cell mediators, such as bradykinin. This occurs in hereditary and acquired angioedema (often associated with B-cell lymphoproliferative diseases), associated with deficiency or dysfunction of the C1 esterase inhibitor, or in angioedema induced by ACE inhibitors.

When cutaneous and/or mucosal symptoms are associated with one or more systemic symptoms (pulmonary, cardio-circulatory, gastro-intestinal, neurological), suggesting an initial anaphylaxis, epinephrine should be prescribed [66].

### Pharmacologic treatment of chronic spontaneous urticaria

#### Antihistamines

These drugs are generally administered in a single daily dose, in order to minimize nocturnal sedation. Bilastine, at a daily dose of 20 mg, levocetirizine (active enantiomer of cetirizine) and desloratadine, both administered at a daily dose of 5 mg, appear to be more effective antihistamines and with less side effects (sedation and interaction with other drugs) compared to 1st generation antihistamines. Other SGA tested in CSU are cetirizine, fexofenadine, loratadine, ebastine and rupatadine. Antihistamines do not modify natural history of CVS, but when symptoms control is achieved must be progressively reduced to indentify the minimal effective dose

If the symptoms are severe or associated with angioedema, the old H1 antihistamines with sedative action, such as hydroxyzine (25 to 50 mg/day) and diphenhydramine (25 mg twice a day) may reduce anxiety and insomnia. Adding a second H1 antihistamine or an H2 histamine in patients with little effect of H1 antihistamine treatment. However, treatments combined with H1 and H2 antihistamines or with two different H1 antihistamines do not seem to provide advantages over monotherapy. At present, it is preferred to increase the dose of a single antihistamine as indicated by the 2017 EEACI/GA2LEN/EDF/WAO guidelines [1] which propose a 4-step therapeutic algorithm. The first step is based on SGA in once daily administration; is symptoms persist after 2 weeks, the second step increases the SGA dosage up to 4 times; if symptoms persist after 2–4 weeks, omalizumab is added; if symptoms persist after 6 months, cyclosporine is added.

*Corticosteroids*

Treatment with corticosteroids is generally not recommended in the treatment of CSU. However, there are cases of in which in the pathogenesis of the disease mediators other than histamine are involved, such as PAF, leukotrienes and other cytokines, and the perivasal infiltrate shows lymphocytes in addition to basophils and eosinophils. These cases respond to a short course of corticosteroids and are generally refractory to treatment with antihistamines [1, 101].

#### Ciclosporin

Ciclosporin has a direct effect on the release of mediators. The efficacy of cyclosporine in combination with second-generation antihistamines has been demonstrated in open-label and double-blind clinical trials [102, 103]. Its use is possible, off-label, in patients who have not responded to antihistamine therapy or to omalizumab. However, the use of this drug as a standard CSU therapy, because of the risk of side effects is not recommended [104].

#### Omalizumab

Omalizumab is a humanized monoclonal antibody derived from recombinant DNA that binds selectively to human IgE [105]. It was initially approved in the USA in 2003 for the therapy of patients with moderate to severe persistent allergic asthma and then in Europe in 2005 for the therapy of allergic asthma resistant to conventional therapies [106] In 2013 Omalizumab was included in the international guidelines as third line for the treatment of patients with CSU resistant to antihistamine therapy and was subsequently officially approved in 2014, both in the United States and Europe, by the drug regulatory agencies [107]. In Italy Omalizumab, at a dose of 300 mg every 4 weeks, was reimbursed by the Health System from August 2015 with the indication of additional therapy in the treatment of CSU in adult and adolescent patients (aged ≥ 12 years) with response inadequate to treatment with antihistamines H1 [108].

Usually the side effects caused by Omalizumab are mild or moderate, but like any drug it can have even serious adverse effects [109]. The most commonly reported side effects are injection site reactions (pain, swelling, itching, erythema), headache and (in children), abdominal pain and fever. Serious (very rare) side effects include anaphylactic shock and systemic lupus.

### Pharmacologic treatment of chronic inducible urticaria

The therapeutic objective consists in the prevention of exposure to the triggering factors and on the symptomatic treatment in order to obtain complete control of the symptoms and clinical signs and to improve the patient’s quality of life [1, 21]. The ideal treatment is in fact based on the elimination of the inducing causes, detected by the accurate investigation of the patient’s clinical history and, if necessary, by appropriate diagnostic tests (Table 4). When the causes are not identifiable, it is in any case indicated to eliminate the factors that can more easily cause a worsening of urticaria: drugs, food additives, foods rich in histamine or its precursors [1]. Regarding the symptomatic pharmacological therapy of CIU, it is based on the use of drugs able to block mast cell mediators (antihistamines H1, antileukotrienes) or to inhibit mast cell activation (omalizumab, cyclosporine) [1]. Currently no large studies are available on the treatment of the main types of CIU, in fact the therapeutic schemes adopted refer to the guidelines for the treatment of CSU [1]. According to current guidelines, second generation H1 antihistamines represent the first line drugs for the treatment of CIU. [1]. When clinical response is unsatisfactory, the successive steps are similar to that for CSU reported above. Of interest, in some cases CIU can be treated through desensitization to trigger factors [110]. This phenomenon has been described for cold urticaria, cholinergic and solar urticaria. Moreover, although rarely, the regress can be spontaneous. Table 6 shows the therapeutic indications for the main forms of CIU.

**Table 6:** Treatment of chronic inducible urticaria

Kind of chronic inducible urticaria	Treatment
Cold urticaria	SGA, desensitization to cold by repeated controlled exposures, in selected cases anakinra or etanercept
Heat urticaria	SGA, possibly associated with H2 antihistamines, omalizumab, desentitization to heat by repeated controlled exposures
Sunlight urticaria	Photoprotective clothing, use of sunscreens, desensitization to UV rays by phototherapy (UVB or UVA), SGA, omalizumab, ciclosporin, intravenous immunoglobulins, afamelanotide
Vibratory angioedema	SGA, avoidance of vibratory stimulus.
Pressure urticaria	SGA, antileukotrienes (possibly in combination), omalizumab
Colinergic urticaria	SGA, omalizumab, scopolamine, danazole
Contact urticaria	SGA, avoidance of the causative agent
Dermographism	SGA up to four daily, omalizumab, ciclosporin
Aquagenic urticaria	SGA, better result if associated with PUVA, barrier creams

*SGA* second generation antihistamines, *UV* ultra-violet

### Urticaria in children

The prevalence of pediatric urticaria varies between 2.1 and 6.7% and appears to be influenced by the atopic constitution [111, 112]. In children, gender differences are less evident and forms of AU are more common; only in 20–30% of cases there is an evolution in chronic urticaria, i.e. lasting more than 6 weeks [113]. It is not uncommon to observe urticaria factitia and contact urticaria, often an expression of food allergy in atopic subjects. Cholinergic urticaria is most commonly seen in adolescents.

The diagnosis of urticaria in childhood does not present particular difficulties and is based on clinical criteria; its clinical presentation is not different from that of the adult. In AU, the hives can more frequently assume a figurative appearance and hemorrhagic aspects and may result in hyperchromic pigmentary outcomes. The association with angioedema is possible in 15% of cases [114]. Pruritus is the characteristic symptom, however in children under 3 years it may be poor or absent. In the case of angioedema located in acroposteous sites, a painful symptomatology may arise at the joints, which may mislead the diagnosis towards a form of acute arthritis. Other possible symptoms are abdominal pain, diarrhea and vomiting. These symptoms may also be present in forms of urticaria vasculitis, but in these cases the hives persist over 24 h and pruritus can be scarce or absent. As in adults, the symptoms and persistence of hives can negatively affect the quality of life of children [115]. Numerous agents can act as triggers [114, 116]. More often than in the adult in the forms of AU it is possible to recognize an infectious etiology [117]. Flu, respiratory or gastrointestinal symptoms may be present 1–2 weeks before or associated with skin manifestations. A number of viral, bacterial, or protozoal agents have been identified as possible causative agents [113, 117]. The second cause of AU is represented by drugs (antibiotics and NSAIDs). However, some studies have shown that children often tolerate suspected drugs as triggers [118].

In case of repeated episodes of AU, a food etiology should be suspected, that must be further researched and confirmed through the compilation of a diary (appearance of symptoms less than an hour after ingestion of a food) and a careful elimination diet. However, food allergies seem to be present in less than 7% of cases of urticaria [1]. The pathogenesis due to immune complexes or autoimmunity seems infrequent in pediatric age [119–121].

The management of urticaria in children is substantially similar to that in adults, as underlined in guidelines and recent reviews [1, 21, 121]. In AU microbiology assessment is not warranted [1, 21, 22]. Specific IgE measurement or skin prick tests are indicated only when history is strongly suggestive of food allergy. In CU possible inflammatory causes may be investigated by blood count with formula, erythrocyte sedimentation rate and C-reactive protein. ASST is not indicated in pediatric age because it does not help in identifying the causes and does not predict the severity or duration of urticaria [1, 21]. Also the search for autoantibodies (anti-thyroid, anti-transglutaminase, antiendomisium, etc.) is justified only in presence of an history clearly suggesting such etiology. All variants of CIU must be assessed by the appropriate tests according to the specific physical. A skin biopsy is justified only in presence of suspected vasculitis. Pediatric urticaria seems to have a favorable course, with no associations with severe diseases. Recent studies reported remission of urticaria in 16% of affected children after 1 year, 38% after 3 years and approximately 50% after 5 years [122].

The same treatment algorithm used for adults may be applied, although the recommendation is weak and based predominantly on a clinical consensus [21]. The first treatment line is represented by SGA [1, 21, 22]. First generation antihistamines negatively impact REM sleep and school learning ability. More frequently than in adults they can also cause paradoxical excitation, constipation and weight gain [123, 124]. The use of first-generation antihistamines should be discouraged both for these known side effects and for the absence of randomized controlled trials supporting efficacy [21, 114, 124]. The most studied SGA are cetirizine, levocetirizine, loratadine, desloratadine, which have evidence of efficacy [21, 114, 124]. Cetirizine, because of a different metabolism in children, should be administered B.I.D. Rupatadine is a SGA with antagonist action against PAF receptors, the efficacy of which was confirmed in controlled trials [125]. Recent studies reported good safety and tolerability in children also for bilastine [126].

If SGA have no effect in controlling pruritus and urticaria, the dose increased up to four times previously reported for adults is not validated in pediatric age [1, 21, 124]. No studies supporting the use of a combination H1 and H2 antihistamines as well as the use of antileukotrienes or ciclosporin are available [127]. If needed, short courses of oral corticosteroids at pediatric dosage for no more than 10 days can be performed. Omalizumab is approved for the treatment of CSU not responding to antihistamines in children aged more than 12 years [128, 129].

### Urticaria in pregnancy

The elevated estrogen-progestin levels as well as the physiological anatomical changes that characterize pregnancy have a significant impact not only on the product of conception but also on the health of the mother. As known, some pathologies such as bronchial asthma, can both improve and worsen during these 9 months, therefore requiring an often-multidisciplinary approach in women who are planning or carrying out a pregnancy. Even some skin diseases show a strong link with pregnancy. High hormone concentrations are known to exacerbate episodes of angioedema in women carrying the XII factor mutation. The same hormonal framework may be the trigger of complex systemic diseases such as SLE which very often has the skin as the first involved organ. Actually, urticaria is not a pathology showing during pregnancy a greater incidence of both early diagnosis and recurrence in women affected already in the pre-conception period [130, 131]. The major problem for patients therefore concerns which therapeutic approach should be preferred, both in acute and in chronic urticaria, in every phase of pregnancy and breastfeeding.

The therapy must at the same time protect the health of the fetus from possible iatrogenic damages, especially during the first trimester, but also safeguard the well-being of the mother. It should not be forgotten that urticaria has a very significant impact on the quality of life and psychological well-being of patients [132], similar to that of atopic dermatitis and psoriasis [133]. Moreover, there are no studies that exclude a potential harmful effect of the high levels of histamine that characterize urticaria [21] as well as of the inflammation and potentially the possible alterations of the coagulation cascade that can be associated with the chronic forms.

The most important issue, however, is that mothers and often even general practitioners perceive the potential harmful effects from drugs as more severe and more frequent than those caused by the disease itself [134–136]. An indicative figure in this regard is the high percentage of pregnant women treated with bronchial asthma [137, 138] and therefore at risk of severe complications of the fetus [139]. It is therefore important to educate physicians and patients about the importance of using the right drugs in situations that make them necessary because even a low level of mother’s health can mean a low level of health of the child.

### Therapy of urticaria in pregnancy

It is important to distinguish between acute and chronic forms and to search for possible triggers and etiological agents. The symptoms of urticaria may be the expression of pathologies that could significantly affect pregnancy, such as food allergies as a risk of anaphylactic reactions [21]. In both acute and chronic urticaria, in addition to the pharmacological approach, it is usually useful to suggest the patient to avoid certain drugs such as the NSAIDs [21]. Concerning foods, in pregnancy prescribing particularly restrictive diets could deprive the child of important nutrients and should therefore be done with particular caution.

Fortunately, a large part of the drugs available to treat acute and CU has proved safe even during pregnancy and lactation. The therapeutic algorithm for this particular patient population is the same one applied to the general population affected by both acute and chronic urticaria, as recommended in the European guidelines [21]. In any case, the risk–benefit ratio for each drug to be used must be evaluated for each patient. During pregnancy, drugs must be used with extreme caution. In this regard, Food and Drug Administration (FDA) foresees 5 risk categories, those belonging to categories A and B being considered safer.

Concerning antihistamines, SGA must be preferred. In particular, the only ones with proven safety during pregnancy are loratadine and cetirizine, classified by FDA as class B drugs. As with SGA in general, they have a low secretion in breast milk and can therefore also be used during breastfeeding. If it is necessary to use first generation antihistamines, the safest are chlorpheniramine and diphenhydramine, tough their concentration in breast milk is higher, leading to a greater risk of sedation of the newborn. To date there are no studies demonstrating the safety of a possible increase in the dosage of antihistamines both before and second-generation during pregnancy [21]. Nevertheless, in the international guidelines, this therapeutic approach was recently approved, even in pregnancy and lactation, which must in any case be implemented with extreme caution [21]. Since these dosages are off-label, it could be prudent—especially in the first trimester of pregnancy—to use antihistamines at increased dosage only in selected cases, using short cycles of corticosteroid therapy to increase the control of urticaria.

Corticosteroid are considered by FDA class C drugs, since prolonged use is associated with the risk of intrauterine infections and prematurity [140] in addition to the well-known side effects such as arterial hypertension and diabetes which can influence the course of pregnancy. The higher incidence of previously reported cleft palate [141] or other congenital defects in women treated with steroids even during the first trimester of gestation has not been confirmed in a recent review of scientific literature [142]. The use of corticosteroids for short periods can be considered relatively safe. The corticosteroid of choice is prednisone, since about 90% of the dose is inactivated by the placental enzyme 11-beta hydroxylase [143]. In fact, EULAR has approved its use in pregnant women suffering from diseases such as lupus or rheumatoid arthritis [142]. Corticosteroid use can reduce a couple’s fertility, because in humans it inhibits the hypothalamic-pituitary–testicular axis and in women it can cause alterations in the ovarian cycle.

As far as biologic agents are concerned, Omalizumab was promoted to class B drug according to the FDA [144]. The use since 2006 in patients suffering from severe asthma has allowed numerous retrospective observational studies that have confirmed its safety also during pregnancy [145]. The case reported in the literature of pregnant women suffering from CU treated with Omalizumab seem to confirm its safety [146–148]. Most authors suggest not starting Omalizumab during pregnancy but not discontinuing its use during conception [149]. Nevertheless, in some of the reported cases of CU therapy with Omalizumab was performed in the gestational age with no report of adverse events or fetal malformations. However, it should be remembered that the use of this drug should be reserved only for particularly serious cases. In fact the data available thus far are very limited, though no adverse effects related to the use of Omalizumab in the progeny of women treated with this biological drug have been reported [150]. Antileukotrienes, particularly montelukast, during pregnancy does not seem to be risky for the health of the fetus [151] so that FDA has classified it as a class B drug. The small number of studies related to its use in pregnant women, however, recommends that its use should be evaluated on a case by case basis [152].

For ciclosporin, no withdrawal is required either during conception or during gestation, as this drug does not appear to reduce fertility [153] and does not cross the placenta, this with no teratogenic effects [154]. Nevertheless, the hypertensive risk, the embryotoxicity described in some murine models, the cases of preterm birth and low birth weight also require very prudent and limit the use of ciclosporin to particularly severe cases [155].

## Conclusions

Urticaria is a term referring to a group of diseases which involve the onset of pruritic wheals, angioedema, or both. Urticaria may be divided into acute and chronic forms. AU is defined as the occurrence of spontaneous wheals, angioedema, or both for < 6 weeks. The diagnostic procedures in patients with AU are fundamentally different from those used in patients with CU. The current recommendations are thus based on a precise formulation of the goals and steps in the recommended diagnostics. Because an underlying cause is rarely detected, a causal and/or curative treatment in not available for the majority of patients. Therefore, symptomatic therapy remains the mainstay of treatment. Modern SGA in licensed doses are the treatment of the first choice. In patients who do not respond adequately to standard dosage of SGA, the dosage should be increase up to four times the standard dosage. Patients who fail to respond adequately even to higher dosages of SGA should be given omalizumab (anti-IgE). More effective and safe therapies should be investigated for patients who do not respond to H1-antihistamines or omalizumab.

## Data Availability

Not applicable.

## Abbreviations

SIAAIC: Italian Society of Allergology, Asthma and Clinical Immunology
SIDAPA: Italian Society of Allergological, Occupational and Environmental Dermatology
AU: Acute urticaria
NSAIDs: Non steroidal anti-inflammatory drugs
CU: Chronic urticaria
ACE: Angiotensin Converting Enzyme
QoL: The quality of life
CRD: Component resolved diagnosis
CIU: Chronic inducible urticaria
HLA: Human Leukocyte Antigen
TPO: Thyroperoxidase
dsDNA: Native DNA
BHRA: Basophil Histamine Release Assay
ASST: Autologous Serum Skin Test
PG: Prostaglandin
LT: Leukotriene
PAF: Platelet activating factor
TNF: Tumor necrosis factor
IL: interleukin
MBP: Major basic protein
PAR: Protease activating receptor
TF: Tissue factor
Th: T helper
GPCR: Type G protein-coupled receptor
MLCK: Myosin light chain kinase
VEGF: Vascular endothelial growth factor
CGRP: Calcitonin gene-related peptide
NO: Nitric oxide
MMP: Metalloproteinase
HRF: Histamine releasing factor
HRQoL: Health related quality of life
PROs: Patient reported outcomes
CU-Q2oL: Chronic Urticaria Quality of Life Questionnaire
CUPP: Chronic urticaria patient perspective
CSVV: Cutaneous small vessel vasculitis
UV: Vasculitic urticaria
NUV: Normocomplementemic vasculitic urticaria
HUV: Hypocomplementemic vasculitic urticaria
SLE: Systemic lupus erythematosus
CAPS: Cryopyrin-associated periodic syndrome
CIAS: Cold induced autoinflammatory syndrome
CINCA: Chronic infantile neurologic, cutaneous and articular syndrome
FDA: Food and Drug Administration
SGA: Second generation antihistamines
UV: Ultra-violet
